# Retraction Note: Oncogenic transformation of human lung bronchial epithelial cells induced by arsenic involves ROS-dependent activation of STAT3-miR-21-PDCD4 mechanism

**DOI:** 10.1038/s41598-019-53858-z

**Published:** 2019-11-15

**Authors:** Poyil Pratheeshkumar, Young-Ok Son, Sasidharan Padmaja Divya, Lei Wang, Zhuo Zhang, Xianglin Shi

**Affiliations:** 1 Center for Research on Environmental Disease, University of Kentucky, 1095 VA Drive, Lexington, KY 40536 USA; 2 Department of Toxicology and Cancer Biology, University of Kentucky, 1095 VA Drive, Lexington, KY 40536 USA

Retraction of: *Scientific Reports* 10.1038/srep37227, published online 23 November 2016

The editors are retracting this article.

During an investigation into an allegation of research misconduct at the University of Kentucky, where the research was conducted, it was determined that the article contains fabricated and/or falsified data^1^.

Specifically, in Figure 2D pSTAT3 control image contains no pixel data. The raw data and metadata for this figure are not available. Figure 3E contains inappropriate scale bars, which use incorrect unit. No scale bars were recorded in the original data; as such the veracity of the scale bars shown cannot be confirmed. pSTAT3 control images in this figure contain no pixel data. Metadata and raw data for figure components in panel 3E are not available.

Since these data are critical to certain main conclusions of the study, the editors retract this article.

Poyil Pratheeshkumar, Lei Wang, and Xianglin Shi do not agree with the retraction. Young-Ok Son, Sasidharan Padmaja Divya, and Zhuo Zhang could not be reached.
